# Notch2/3-DLL4 interaction in urothelial cancer cell lines supports a tumorigenic role of Notch signaling pathways in bladder carcinoma

**DOI:** 10.1371/journal.pone.0317709

**Published:** 2025-02-14

**Authors:** Chuan Zhang, Annett Weimann, Jens-Uwe Stolzenburg, Jochen Neuhaus, Mandy Berndt-Paetz

**Affiliations:** 1 Department of Urology, Research Laboratories, Leipzig University, Leipzig, Germany; 2 Department of Urology, Chengdu Fifth People’s Hospital affiliated to Chengdu University of T.C.M., Chengdu, China; 3 Department of Urology, University Hospital Leipzig, Leipzig, Germany; University of California Santa Barbara, UNITED STATES OF AMERICA

## Abstract

**Introduction:**

The Notch pathway plays an important role in many aspects of cancer biology and acts in a dichotomous way in bladder cancer. The mechanisms behind this behavior are still elusive. Here, we analyzed DLL4 and Notch receptor expression, interaction and downstream signaling in human bladder cancer cells.

**Materials and methods:**

The expression levels of Notch pathway components (Notch1-4, DLL4, HES1, HEY1) were assessed in papillary (G1: RT-4) and non-papillary bladder cancer cell lines (G2-G4: RT-112, 647-V, T-24, KU-19-19, CAL-29) by qRT-PCR and immunofluorescence. Expression data were validated by analyzing data from open-source databases (CCLE; TCGA). The endogeneous interactions of Notch2/Notch3 receptors and the ligand DLL4 were studied by in situ proximity ligation assay. Activation of canonical Notch signaling was evaluated by stimulation with recombinant DLL4 protein.

**Results:**

All Notch targets were expressed, with Notch2 and Notch3 showing the highest expression levels. Endogeneous interactions between Notch2/3 and DLL4 were detected in all BCa cell lines. Amounts of Notch2/3-DLL4 complexes were high in RT-112 and CAL-29, while RT-4/647-V showed moderate and T-24, KU-19-19 low abundance. Proportion of (peri-) nuclear interaction complexes correlated negatively with Notch downstream targets. DLL4 stimulation resulted in canonical Notch pathway activation and increased tumor cell viability and proliferation in RT-4, 647-V, T-24 and KU-19-19 cells.

**Discussion:**

The Notch signaling pathway can discriminate between different receptors and may play an essential role in the progression of bladder carcinoma. We demonstrated for the first time direct interactions between DLL4 and Notch2/3 associated to activation of canonical downstream Notch signaling and increased tumor cell behavior in human bladder cancer cells. Our data support the view that the Notch2/3-DLL4 axis plays an oncogenic role in bladder cancer. Further analyses of Notch signaling in bladder cancer can promote the development of tailored anti-DLL4/Notch bladder cancer therapies in the future.

## Introduction

Notch signaling is a highly conserved ligand-receptor signaling pathway, playing an essential role in maintaining stem cells, cell proliferation, survival, apoptosis, and differentiation in many normal tissues and organs [1–3]. Over the past two decades, evidence accumulated, revealing that the Notch pathway is associated with various human cancers, for example, breast cancer [4–6], lung cancer [7], colorectal cancer [8,9], pancreatic cancer [10] and prostate cancer [11]. Furthermore, the Notch signaling pathway is also a promising target for cancer therapy [3,12]. In the past six years, the Notch signaling pathway has received considerable attention in BCa [13].

To date, four Notch receptor genes (Notch1, Notch2, Notch3 and Notch4) and five ligand genes (DLL1, DLL3 and DLL4, JAG1 and JAG2) are known in the human genome [13]. Notch receptors and its ligand genes encode single transmembrane proteins, and their interaction can activate canonical or non-canonical Notch signaling pathways [12,14,15]. Ligand binding either results in releasing the intracellular domain of Notch (NICD), which then travels to the nucleus and initiates various transcriptional programs affecting various cellular functions (canonical pathway), or directly interacts with downstream effectors (non-canonical pathway) including mTOR, AKT, PI3K, Wnt, NF-kB, or HIF-1α to activate associated pathways at either the cytoplasmic and/or nuclear level independently of CSL (CBF1/RBPJk) [12–15]. However, both canonical and non-canonical Notch signaling contributes to regulate cell survival, immunity, tumor angiogenesis, epithelial-mesenchymal transition (EMT), or drug resistance [15–18]

Indeed, receptor-specific, and ligand-specific effects of Notch signaling have been investigated in BCa. For example, Rampias et al. described that Notch1, Notch2 and Notch3 act as tumor suppressors in BCa [19]. Hayashi et al. reported the oncogenic role of Notch2 in BCa [20]. Zhang et al. affirmed that Notch3 was significantly overexpressed in BCa indicating a tumor promoting action of Notch3 [21]. Patel described that DLL4 was significantly upregulated in BCa, and the expression of DLL4 was associated with vascular differentiation in BCa [22]. Apparent contradictions among precious studies were described on expression levels, and dichotomous roles of different Notch receptors and ligands were reported, being either oncogene or tumor-suppressor.

All Notch members coexist in BCa, but possibly do not serve the same function [13]. Different ligands can activate different Notch pathways, yielding different results [23]. So far, information about the role of Notch signaling in BCa is limited. Our recent bioinformatics study documented that Notch2, Notch3 and DLL4 are potential drivers of Notch signaling in BCa, supporting the view that Notch signaling plays a crucial role in the progression and development of BCa via modulating the cell cycle, regulating the tumor immunity, and direct or indirect interactions with well-known cancer-related pathways such as p53, TSC/mTOR, RAS/MAPK, and PI3K/AKT [24]. Therefore, we performed the present study to verify the outcomes of the bioinformatic findings by analyzing the oncogenic role of DLL4 and Notch receptors in vitro. We hypothized that DLL4 is directly associated to Notch2 and/or Notch3 receptors resulting in Notch downstream activation and increased tumor cell proliferation. Gene and protein expression of Notch pathway components (Notch receptors: Notch1-4 and ligand: DLL4) was quantified in a panel of BCa cell lines (RT-4, RT-112, 647-V, T-24, KU-19-19, CAL-29). The relevance of our expression results was validated by analyzing data from open-source databases. We further investigated endogeneous ligand-receptor interactions indicating cis-activation (within the same cell) of Notch signaling in BCa cells. Potential trans-activation of Notch signaling via the DLL4 – Notch2/3 axis was evaluated by in vitro stimulation of BCa cells with recombinant human DLL4 protein.

## Materials and methods

### Cell culture

Six human BCa cell lines of various origin (Table 1) were obtained from the Leibniz Institute DSMZ (German collection of microorganisms and cell cultures GmbH, Braunschweig, Germany).

**Table 1:** Characteristics of the used bladder cancer cell lines.

Cell line	Acc.-No	Characteristics		
Origin (gender, age, stage)	Invasiveness	Grade
RT-4	ACC-412	male, 63y, T2	NMIBCa	1
RT-112	ACC-418	female, n.n., n.n.	NMIBCa	2
647-V	ACC-414	male, n.n., n.n.	n.n.	2
T-24	ACC-376	female, 81y, n.n.	MIBCa	3
KU-19-19	ACC-395	male, 76y, T3	MIBCa	3
CAL-29	ACC-515	female, 80y, T2	MIBCa	4

Acc.-No = accession number (DSMZ); MIBCa = muscle-invasive bladder cancer; NMIBCa = non-muscle-invasive bladder cancer.

All BCa cell lines were cultured in RPMI1640 medium supplemented with 10% FBS (Biochrom, Berlin, Germany) and 1% CTS™ GlutaMAX™-I Supplement (Thermo Fisher Scientific, Dreieich, Germany). Cells were maintained at 37°C and 5% CO2 in humidified atmosphere. To obtain single-cell suspension of each cell line, Accutase cell detachment solution (PanBiotech, Aidenbach, Germany) was applied.

### RNA extraction and qRT-PCR

For analyzing mRNA expression of Notch pathway components (NOTCH1-4, DLL4, HES1/HEY1) total RNA was isolated with the RNeasy Plus Micro Kit (Cat.-No.: 74034, Qiagen, Hilden, Germany) according to the manufactory manual. Synthesis of cDNA was performed using the Maxima First Strand cDNA Synthesis Kit (Cat.-No.: K1641, Thermo Fisher Scientific, Dreieich, Germany). Quantitative PCR was done on a qTower^3^ qPCR cycler (Analytik Jena, Jena, Germany) using SYBR-Green quantitative PCR Mastermix (Cat.-No.: K0221, Thermo Fisher Scientific, Dreieich, Germany) and custom primers (MWG-Biotech, Ebersberg, Germany) (Table 2). The constantly expressed human actin beta (βACT) served as reference gene. Comparable efficiencies of primers used for target and housekeeping gene amplification were determined by analyzing serial cDNA dilutions. Detailed step-by-step protocols are provided in S1 Methods.

**Table 2:** List of primers for detection of Notch pathway components by qRT-PCR.

Gene name	Marker	Acc.-No.	Sequence (5’ - 3’)	Annealing (°C)	Product size (bp)
Notch1	Notch1 transmembrane receptor	NM_017617	f - TGGGACCAACTGTGACATCA	57 °C	156
			r - GCACACTCGTTGATGTTGGT		
Notch2	Notch2 transmembrane receptor	NM_001200001	f - AACACTTACAACTGCCGCTG	57 °C	162
			r - ACTCCAGCCGTTGACACATA		
Notch3	Notch3 transmembrane receptor	NM_000435	f - CAGCTTCTCATGTGCCTGTG	59 °C	245
			r - CACAGTCGTAGCGGTTGATG		
Notch4	Notch4 transmembrane receptor	NM_004557	f - CAACCCTGTCACAACCATGG	59 °C	224
			r - ATCTCCACCTCACACCACTG		
DLL4	Delta like canonical Notch ligand 4	NM_019074	f - CCCTTCAATTTCACCTGGCC	58 °C	185
			r - GTGCTGGTTTGCTCATCCAA		
HES1	Hes family basic helix-loop-helix transcription factor 1	NM_005524.4	f - TGAGCCAGCTGAAAACACTG	57.5 °C	158
			r - GTCACCTCGTTCATGCACTC		
HEY1	Hes related family basic helix-loop-helix transcription factor with YRPW motif 1	NM_01228.4	f - TCTGAGCTGAGAAGGCTGGT	57.5 °C	200
			r - CGAAATCCCAAACTCCGATA		
βACT	Actin beta	NM_001101.5	f - CCTCTATGCCAACACAGTGC	59 °C	365
			r - CCTGCTTGCTGATCCACATC		

Acc.-No = accession number; bp = base pairs.

### Immunofluorescence

Cells were cultured on collagen A-coated 13 mm-coverslips for 24 h. Cells were fixed with ice-cold methanol and permeabilized using a DMSO/Triton-X-100 mixture. Primary antibodies (Table 3) were incubated overnight at 4°C. Technical negative control staining was performed by omitting the primary antibody. Alexa Fluor 488^®^- and Alexa Fluor 555^®^-coupled secondary antibodies (Thermo Fisher Scientific, Dreieich, Germany) diluted in TBS (1 h, room temperature), were used for indirect immunofluorescence of target proteins. Nuclei were labeled with TO-PRO-3 iodide (Thermo Fisher Scientific, Dreieich, Germany). A detailed step-by-step protocol is provided in S1 Methods. Samples were analyzed by confocal laser scanning microscopy (LSM 800, Carl Zeiss, Jena, Germany). Fluorescence intensities of Alexa Fluor 555^®^-labeled targets (553 nm excitation/568 nm emission) were quantified in CK AE1/AE3-positive cells (region of interests, ROIs) using ImageJ [25].

**Table 3:** Overview on antibodies used for detection of Notch pathway components by immunofluorescence and PLA.

Antigen	Specificity	Host	Source	Cat.-No.	Dilution
Notch1	Notch1 transmembrane receptor	Rb	Cell Signaling Technology Inc., Danvers, MA, USA	#3608	1:200
Notch2	Notch2 transmembrane receptor	Rb	Abcam, Cambridge, UK	ab8926	1:500
Notch3	Notch3 transmembrane receptor	Goat	Novus Biologicals, Abingdon, UK	NB100-2414	1:200
Notch4	Notch4 transmembrane receptor	Rb	Thermo Scientific, Dreieich, Germany	PA5-40587	1:100
DLL4	Delta like canonical Notch ligand 4	Ms	Thermo Scientific, Dreieich, Germany	MA5-17069	1:200
HES1	Hes family basic helix-loop-helix transcription factor 1	Rb	antibodies-online, Aachen, Germany	ABIN2779597	1:200
HEY1	Hes related family basic helix-loop-helix transcription factor with YRPW motif 1	Rb	antibodies-online, Aachen, Germany	ABIN182593	1:200
Cytokeratin cocktail (AE1/AE3)	Epithelial cells	Ms	Biogenex, Fremont, CA, USA	MU071-UC	1:200

Rb = rabbit; Go = goat.

### Proximity ligation assay

PLA was used to detect interactions of DLL4 with Notch receptors Notch2 and Notch3. Prior to PLA staining, cells were cultured on collagen A-coated 13 mm-coverslips for 24 h. Cells were fixed with 3.7% formaldehyde for 30 min at 4°C. Permeabilization and primary antibody treatment was performed as described above. PLA was performed according to the manufacturer’’s instructions. Duolink™ In Situ PLA probes (Anti-Rabbit PLUS, Cat.-No.: DUO92002; Anti-Mouse MINUS, Cat.-No.: DUO92004; Anti-Goat MINUS, Cat.-No.: DUO92006; Sigma-Aldrich, Munich, Germany) were applied to the coverslips for 1 h at 37°C. Following several wash steps, samples were incubated with the ligation mix for 30 min at 37°C and with the Cy3 amplification mix (Duolink™ In Situ Detection Reagents Orange, Cat.-No.: DUO92007, Sigma-Aldrich, Munich, Germany) for 100 min at 37°C. Technical negative control staining was performed by incubation with only one primary antibody, followed by treatment with PLA probe mixture and PLA detection reagents. Alexa Fluor^®^ 488-labeled wheat germ agglutinin (WGA) was used for visualization of cells and nuclei were stained with DAPI contained in the Duolink™ In Situ Mounting Medium (Cat.-No.: DUO82040, Sigma-Aldrich, Munich, Germany). A detailed step-by-step protocol is provided in S1 Methods. Samples were analyzed using a LSM 800 confocal laser scanning microscope (Carl Zeiss, Jena, Germany). Images for quantification were acquired at constant settings (40 x/ 0.95 NA objective); pinhole of the 561 nm laser line was appropriately adjusted to 59 µm (1.2 AU; 1.4 µm optical section) to capture all PLA signals. The analysis workflow was described previously [26]. Quantification of PLA signals was done at single cell level by particle analysis using a self-written ImageJ Macro. WGA-positive cells (or DAPI-positive nuclei) served as regions of interest (ROI). Amounts of particles were normalized to ROI sizes. Approximately 100 to 150 single cells were analyzed per PLA and cell line. Images to verify subcellular localization of PLA signals were acquired at high resolution using a 63 x/ 1.4 NA oil immersion objective (pinhole: 36 µm; 0.68 AU; 0.7 µm optical section).

### DLL4 ligand treatment

Recombinant human DLL4 (Cat.-No.: #A42513) was purchased from ThermoFisher Scientific (Dreieich, Germany). DLL4 stock solution (100 µg/ml) was prepared in sterile double-distilled water. Prior to treatment, BCa cells were cultured for 24 h in suitable culture formats (coverslips: PLA; 35 mm dishes: qRT-PCR; 96 well plates: functional cell-based assays). Samples were then treated with DLL4 (0, 250, 500, 1000 ng/ml; diluted in culture medium). Distilled water diluted in medium served as negative control (vehicle control). Detection of NOTCH2/3-DLL4 interactions was performed after 15 min DLL4 by PLA. Expression of canonical Notch effectors (HES1/HEY1) was measured by qRT-PCR after 6 and 16 h of DLL4 stimulation. Percentage of viable cells was determined after 72 h using CellTiter-Glo^®^ Luminescence Cell Viability Assay (Cat.-No.: G7570, Promega, Mannheim, Germany). Amounts of cells in proliferation were detected using CellTiter 96^®^ Non-Radioactive Cell Proliferation Assay (Cat.-No.: G4000, Promega, Mannheim, Germany). Cell-based assays were measured on a SpectraMax M5 microplate reader (Molecular Devices, Sunnyvale, USA). Detailed step-by-step protocols for viability and proliferation assays are provided in S1 Methods. Migration was evaluated by live-cell imaging using the Incucyte^®^ Live-Cell Analysis System (Sartorius, Epsom, UK).

### Statistical analysis

Statistical analyses of experiments were performed using Prism 10.0.2 statistical software (GraphPad Software Inc., La Jolla, CA, USA). Bar diagrams present the means +  standard deviation from at least three independent experiments. Gaussian distribution of values was tested by the D’Agostino-Pearson omnibus normality test. Statistical differences of expression levels were analyzed by One-way ANOVA (Tukey’s multiple comparisons test). Statistical differences between DLL4-treated and untreated samples were analyzed by One-way ANOVA (Dunnett’s multiple comparisons test) or Mann-Whitney test, depending on the number of test groups. Covariances were analyzed using Pearson’s correlation coefficient. P ≤  0.05 was considered statistically significant.

## Results

BCa cell lines were characterized regarding their expression of Notch receptors, DLL4 ligand and canonical Notch downstream targets. Endogeneous interactions of Notch2/3 with DLL4 were analyzed by in situ PLA. Further, we evaluated the response of BCa cells to DLL4 ligand stimulation.

### Expression of Notch receptors and DLL4

**Fig 1:**
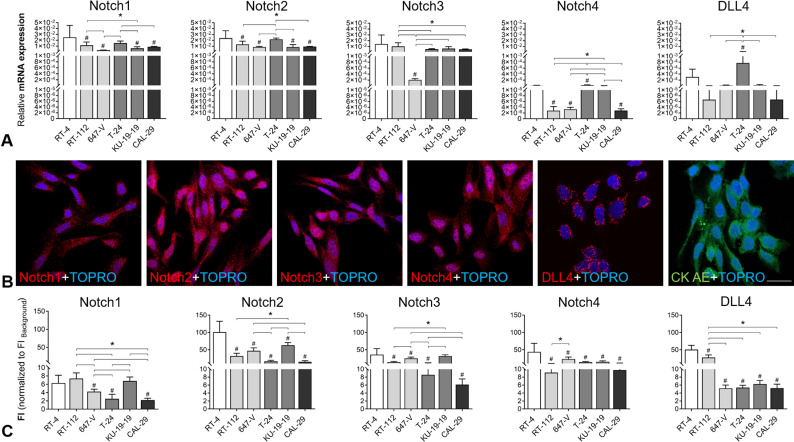
Expression of Notch receptors and DLL4 ligand in BCa cell lines. (A) Relative mRNA expression (normalized to housekeeping gene β-actin) of target genes by qRT-PCR in different BCa cell lines; # significance versus non-invasive, papillary RT-4 cells (G1); *  significance between G2-G4 cell lines; p ≤  0.05, One-way ANOVA, Tukey’s multiple comparisons test, mean +  SD, n = 3. (B,C) Protein expression of targets by immunofluorescence in different cell lines. (B) Representative immunofluorescence images of Notch1, Notch2, Notch3, Notch4 and DLL4 staining, exemplarily shown for RT-4 cells; scale bar: 20 µm. (C) Quantification of mean fluorescence intensity in CK AE-positive ROIs; # significance versus non-invasive, papillary RT-4 cells (G1); *  significance between G2-G4 cell lines; p ≤ 0.05, One-way ANOVA, Tukey’s multiple comparisons test, mean +  SD, n = 3.

We analyzed the expression patterns of Notch receptors (Notch1-4) and DLL4 in various BCa cell lines (papillary: RT-4 (non-invasive, grade 1) vs. non-papillary: RT-112, 647-V, T-24, KU-19-19, CAL-29 (non-invasive/invasive, grade 1-4) by qRT-PCR and immunofluorescence (Fig 1). Target gene expression was normalized to actin beta expression. Protein expression was determined by quantification of target fluorescence intensity in CK AE-positive cells.

All the targets genes were detected with different expression levels in various BCa cell lines.

The comparison of mRNA and protein expression data showed some major differences, which could be due to post-translational modifications. However, Notch2 and Notch3 showed highest expression both on mRNA and protein level, especially in RT-4, 647-V and KU-19-19 cells. When comparing papillary with non-papillary cell lines, the papillary RT-4 cell line showed significantly enhanced expression of all Notch receptors and DLL4 ligand. The exact quantitative data and detailed significances are provided in S1 Table.

To validate the relevance of the cell culture findings, we compared our results with expression data from open-source databases (S1 Fig). Expression level data of Notch1-4 and DLL4 in cell lines were downloaded from the CCLE (cancer cell line encyclopedia) database. As shown in S1A and S1B Fig, our mRNA expression levels of Notch1-4 and DLL4 are roughly consistent with CCLE data. Only T-24 showed major deviations regarding the expression of Notch3 and DLL4. In general, mainly Notch1-3 are abundant, while Notch4 was expressed at low levels in all BCa cell lines. In addition, gene expression data of the BCa cell lines (downloaded from CCLE) were correlated to primary BCa tissue expression data using the TCGA database (previously published [24]). This analysis revealed a moderate to high median correlation coefficient ranging from 0.58 to 0.75 (S1C and S1D Fig), suggesting that the examined cell lines quite closely represent the characteristics of primary tumor tissue.

Cellular localization of the highly expressed Notch2/3 and the DLL4 ligand was investigated by double immunofluorescence labeling (S2 and S3 Figs). While Notch2/3 and DLL4 were diffusely distributed throughout the cytoplasm of 647-V cells, Notch proteins in RT-112 showed a cytoplasmic (DLL4, Notch3), perinuclear (DLL4, Notch2) or nuclear (Notch3) localization. In RT-4, DLL4 showed a major cytoplasmic localization; Notch2 was abundant in the cytoplasm and the perinuclear region; and Notch3 was detected in the cytoplasm or at the cell membrane (S2 Fig). While Notch2/3 and DLL4 were diffusely distributed throughout KU-19-19 cells, Notch proteins showed a predominantly perinuclear or nuclear localization in T-24 and CAL-29 cells (S3 Fig).

### Endogeneous NOTCH2/3-DLL4 interactions

**Fig 2:**
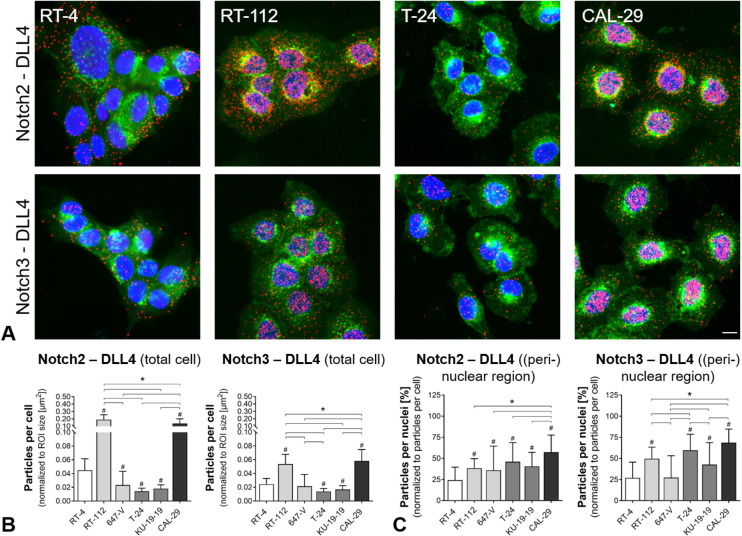
Interactions between Notch2 and Notch3 receptors and DLL4 ligand in BCa cell lines. (A) Visualization of co-localization between DLL4 and Notch2/3 receptors by PLA, exemplarily shown for each grade. Prominent fluorescent spots indicate receptor-ligand interactions. Notch2/3-DLL4 complexes were frequently observed in the (peri-) nuclear region, especially in RT-112, T-24 and CAL-29 cells. Notch2/3 complexes (red); plasma membrane (green); nuclei (blue). Scale bar: 10 µm (B,C) Quantification of PLA signal density by particle analyses at single cell level. (B) Sums of particles were normalized to the area of cells (ROIs). The amount of Notch2/3-DLL4 complexes was significantly higher in RT-112 and CAL-29 cells (vs. RT-4, 647-V T-24, KU-19-19). Low grade RT-4 and 647-V (G2) showed moderate Notch2/3-DLL4 levels, while T-24 and KU-19-19 exhibited equally low PLA signal densities. (C) Percentage of interactions complexes with (peri-) nuclear localization. PLA signals visible within DAPI-positive ROIs were normalized to total amount of signals. *  Significance between G2-G4 cell lines; **p** ≤  0.05, One-way ANOVA, Tukey’s multiple comparisons test; mean +  SD, n = 3.

Our recent bioinformatics study supported the hypothesis of an oncogenic role of Notch2, Notch3 and DLL4 [24]. Based on our previous work and the expression analyses, we established PLA to investigate the endogenous interactions between the highly abundant Notch2/3 and DLL4 (Fig 2).

Application of primary antibodies against Notch2/3 and DLL4 in PLA allowed visualization of DLL4 co-localized with Notch2 and Notch3. Representative images of highly fluorescent, red dots showing Notch interaction complexes are supplied in Fig 2A.

Intriguingly, a robust PLA signal was detected in all BCa cell lines. Numbers of Notch2/3-DLL4 interaction complexes were much enhanced in RT-112 and CAL-29, while RT-4 showed moderate abundance of complexes. Amounts of Notch2/3-DLL4 signals were significantly lower in T-24 and KU-19-19; there was no difference between the grade 3 cell lines (Fig 2B). Exact quantitative data and significances are presented in S1 Table. The total number of complexes was independent from cell line grading for both target interactions (S4A Fig). Interestingly, cell lines showed differences in the localization of complexes. The papillary low-grade RT-4, for example, showed cytoplasmic PLA signals, while high-grade CAL-29 present a predominantly nuclear localization in 1.4 µm optical sections (Fig 2A and C). Increased optical sectioning sharpness using a 63 x/1.4 NA oil immersion objective (pinhole: 36 µm; 0.68 AU; 0.7 µm optical section) verified a predominately cytoplasmic/ membranous localization of Notch2/3-DLL4 in RT-4, while Notch2/3 was predominately found in the perinuclear region of CAL-29 cells (S5A Fig). In this regard, the percentage of (peri-) nuclear Notch2/3-DLL4 complexes correlated significantly with the cell line grading (S4B Fig). Staining negative controls were almost free of PLA signals (S5B Fig).

### Correlation of the Notch2/3 axes with canonical downstream targets

**Fig 3:**
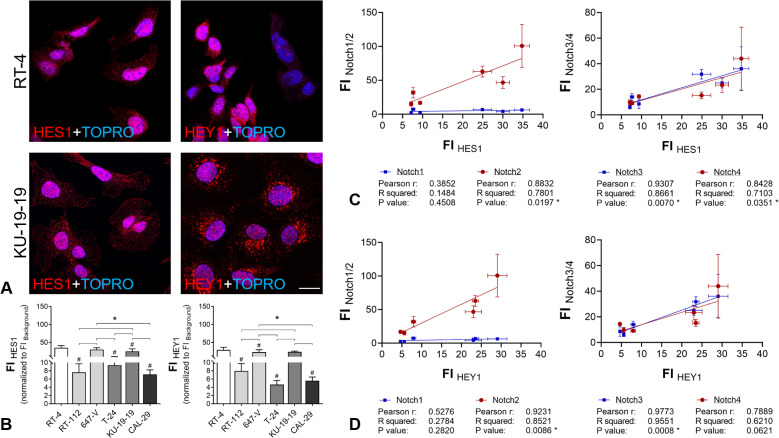
Downstream effectors of canonical Notch in BCa cell lines. (A,B) Protein expression of canonical downstream molecules by immunofluorescence in different cell lines. (A) Representative immunofluorescence images of HES1 and HEY1 staining, exemplarily shown for RT-4 and KU-19-19 cells; scale bar: 20 µm. (B) Quantification of mean fluorescence intensity in CK AE-positive ROIs; # significance versus non-invasive, papillary RT-4 cells (G1); *  significance between G2-G4 cell lines; p ≤  0.05, One-way ANOVA, Tukey’s multiple comparisons test, mean +  SD, n = 3. (C,D) Correlation of HES1 and HEY1 with Notch receptors. (C) Fluorescence intensity (FI) of HES1 show significant correlation to FI of Notch 2, Notch3 and Notch4; (D) HEY1 is significantly related to Notch2 and Notch3; *  p ≤  0.05; Pearson correlation analysis; n = 3.

To gain insights into canonical downstream Notch signaling in the tested cell lines, we analyzed the protein expression of the two well-known downstream targets of the canonical Notch signal pathway, transcription factor HES1 (hairy and enhancer of split-1) and the transcriptional repressor HEY1 (Hairy/enhancer-of-split related with YRPW motif protein 1) by immunofluorescence analysis (Fig 3).

Staining was mainly observed in the cytoplasm and perinuclear region (Fig 3A). Fluorescence intensity of HES1 and HEY 1 was significantly enhanced in RT-4, 647-V and KU-19-19 (Fig 3B). We found significant correlations of both, HES1 and HEY1 with Notch2 and Notch3, while Notch1 and Notch4 showed no correlation with downstream targets (Fig 3C and D). Interestingly, total amounts of Notch2/3-DLL4 interaction complexes showed no correlation with HES1/HEY1 protein expression (S6A Fig). However, the proportion of (peri-) nuclear Notch3-DLL4 complexes correlated negatively with HES1/HEY1 expression (HES1: p = 0.0191; HEY: p = 0.0024) (S6B Fig).

### DLL4-dependent Notch pathway activation

**Fig 4:**
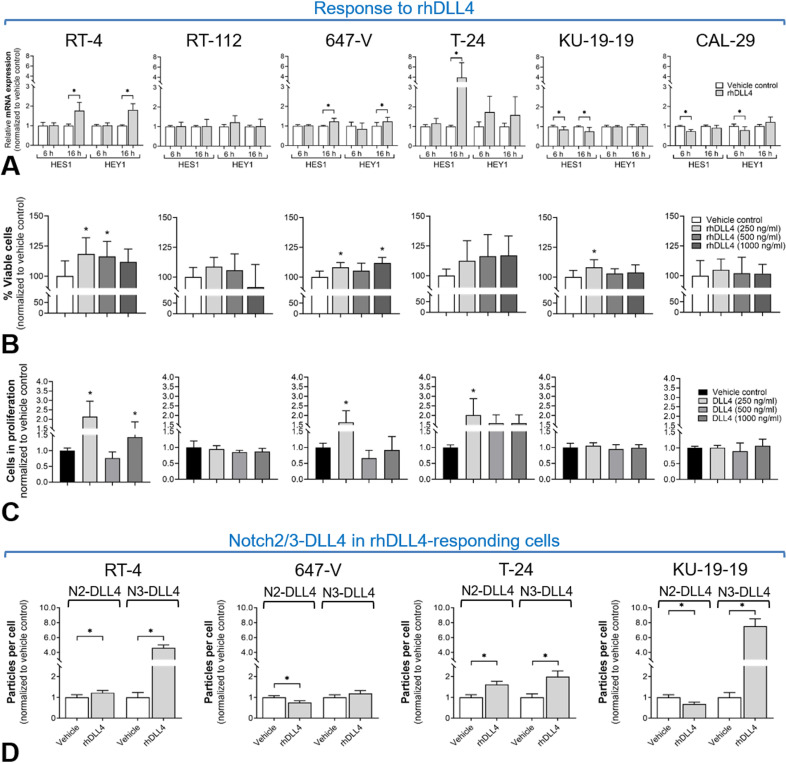
Response to recombinant human DLL4 (rhDLL4) in BCa cells. (A-C) Screening on canonical Notch pathway activation by quantification of HES1 and HEY1 gene expression after 6, 16 h of 250 ng/ml rhDLL4 (A); and quantification of cell viability (B) and proliferation (C) after 72 h of rhDLL4 incubation using different concentrations. RT-4, 647-V and T-24 exhibited increases in HES1/HEY1 gene expression and enhanced cell viability/proliferation after rhDLL4, while RT-112 and CAL-29 showed no DLL4 response in both readouts; *  significant differences to vehicle controls, p ≤  0.05, Tukey’s multiple comparisons test; mean +  SD. (C) Notch2/Notch3-DLL4 receptor interactions in rhDLL4-responding cells. Quantification of PLA signal density by particle analyses after 15 min stimulation with 250 ng/ml rhDLL4. Incubation with DLL4 resulted in significantly enhanced interaction complexes in RT-4, T-24 and KU-19-19, especially for Notch3-DLL4; *  significant differences to vehicle controls, p ≤  0.05, Mann-Whitney test; mean +  SD.

We stimulated BCa cells with recombinant human DLL4 (rhDLL4) to simulate DLL4-mediated Notch trans-activation. We evaluated Notch2/3-DLL4 coupling, activation of canonical downstream targets and pro-tumorigenic cell behavior (survival, proliferation, migration) (Fig 4). Exact quantitative data and significances are presented in S1 Table. Treatment with DLL4 resulted in significantly enhanced HES1/HEY1 downstream effector expression in RT-4, 647-V and T-24 after 16 h. In accordance, significantly increased cell viability and proliferation was detected in these cell lines after 72 h of DLL4 stimulation. KU-19-19 cells showed contradictory results with reduced HES1/HEY1 effector expression and constant proliferation levels, but increased cell viability at 250 ng/ml rhDLL4 (Fig 4A–C). Live-cell imaging of scratch wound assays indicated increased migration in RT-4, T-24 and KU-19-19 by rhDLL4, although differences were not significant due to high standard variations (S7 Fig). RT-112 and CAL-29, which had very high levels of total endogeneous Notch2/3-DLL4 complexes (Fig 2), showed no response to rhDLL4 regarding viability, proliferation and migration (Figs 4A–C and S7). Binding of the DLL4 to Notch2/Notch3 was investigated in the responding cell lines by determination of Notch2/3-DLL4 close proximity via PLA. Using this technique, we were able to detect an increase in interaction complexes in RT-4, T-24 and KU-19-19 after 15 minutes of DLL4 incubation compared to the vehicle control, especially for Notch3-DLL4. In the 647-V cells, rhDLL4 treatment reduced Notch2-DLL4 signals and the number of Notch3-DLL4 interactions remained unchanged. Interestingly, in KU-19-19 Notch2-DLL4 signals were also reduced, however, those cells showed a pronounced increase in Notch3-DLL4 interactions (Fig 4C).

## Discussion

The Notch pathway seems to play a crucial role in bladder cancer. Accumulating literature dominated that Notch signaling can either have oncogenic or suppressive function in BCa depending on the receptor-specific and ligand-specific effects [13]. Our recent bioinformatics study analyzed bulk RNA expression data of Notch pathway components in primary tissue and supported the hypothesis of an oncogenic role of Notch2 and Notch3, but also DLL4 in BCa [24]. However, each compartment of the tumor microenvironment (tumor cells, fibroblasts, endothelial cells, immune cells) expresses a variety of Notch ligands and receptors [27]. Notch signaling is either induced at cell-cell contacts between a signal-sending and a signal-receiving cell (trans-activation), or within the same cell (cis-activation). Endothelium-expressed Notch ligands can trans-activate Notch signaling in adjacent cancer cells leading to increased tumorigenicity. For example, DLL4 expressed by endothelial cells was shown to activate Notch signaling via Notch3 in T cell acute lymphatic leukemia [28] and via Notch1 in colorectal cancer [29]. In another study, JAG1-positive fibroblasts were shown to interact with Notch3 in breast cancer cells to regulate resistance to chemotherapy [30]. In addition to the well-documented Notch crosstalk between endothelial cells/fibroblasts and tumor cells, Notch interactions between tumor cells were also recently demonstrated. In this study, Notch signaling induces differentiation of neighboring cancer cells resulting in intratumoral heterogeneity [31]. Notch crosstalk between BCa tumor cells has not yet been investigated. Here, we analyzed Notch1-4 receptor and DLL4 ligand expression at mRNA and protein level in six BCa cell lines. All targets were expressed, with Notch2 and Notch3 showing the highest expression levels, especially in papillary RT-4 cells but also in non-papillary 647-V and KU-19-19. This is in line with expression data from previous reports extracted from the open-source database CCLE. Further, bioinformatic data from TCGA database suggested that the used cell lines well-matched with primary tumors.

Based on our previous work and the expression analyses showing high abundance of Notch2/3, we investigated the endogenous interactions of DLL4 and Notch2/3 in BCa cell lines by PLA. We were able to visualize the close proximity of DLL4 and Notch2/3 suggesting cis-activation via direct interaction of those targets in BCa cells. Cis-activation of Notch2 by DLL4 has already been demonstrated in cultured colorectal adenocarcinoma cells [32]. Amounts of Notch2/3-DLL4 complexes were especially high in RT-112 and CAL-29, while RT-4 and 647-V showed moderate and T-24/KU-19-19 low abundance. Interestingly, cell lines showed differences in the localization of DLL4, Notch2/3 and their interaction complexes. While Notch2/3 and DLL4 were more or less diffusely distributed throughout the cytoplasm of RT-4, 647-V and KU-19-19, Notch proteins in RT-112, T-24 and CAL-29 showed a cytoplasmic (DLL4, Notch3), perinuclear (DLL4, Notch2) and/or nuclear localization (Notch3). It has already been shown that Notch proteins are continuously redistributed to different subcellular compartments depending on the cellular context [33]. In addition, non-papillary cell lines (especially RT-112, T-24, CAL-29) frequently showed perinuclear localization of interaction complexes, which correlated positively with cell line grading. However, whether the perinuclear PLA signals are actually functionally active interaction complexes or whether the signal is merely a result of the spatial proximity of the target proteins remains unresolved at this point. DLL4 was identified as ligand of Notch1 in hepatocellular carcinoma [34], Notch4 in glioblastoma [35] and Notch3 in melanoma [36]. To the best knowledge of the authors, this is the first study indicating crosstalk between Notch2/3 and DLL4 in BCa cells.

The canonical Notch downstream targets HES1 and HEY1 act as transcriptional regulators. The strong positive correlation with Notch3 and Notch3 expression provide further evidence that these receptors play an essential role in mediating canonical Notch signaling in BCa. Interestingly, total Notch2/3-DLL4 amounts showed no correlation with downstream targets, but proportion of nuclear Notch3-DLL4 correlated negatively with HES1/HEY1 expression. This is in accordance with the hypothesis, that only membraneous or cytoplasmic receptors are actively involved in Notch signaling [33]. Trans-activation of canonical Notch via binding of exogeneous DLL4 to Notch2/3 was further investigated by stimulation experiments. Application of rhDLL4 led to a response in four of six cell lines (RT-4, 647-V, T-24, KU-19-19) in terms of enhanced HES1/HEY1 expression, and/or increased cell viability and proliferation. Recently, it was found that Notch signaling promotes BCa proliferation and metastasis via activation of the Notch2/HEY1 axis [37]. In our study, we were able to detect increased Notch2/3-DLL4 interaction complexes in RT-4, T-24 and KU-19-19 after DLL4 treatment, especially for Notch3-DLL4. An oncogenic role of Notch2 and Notch3 in BCa was previously suggested [20,21]. In addition, DLL4 was recently identified as a an upstream inducer of the Notch3/WNT5B axis mediating bidirectional prometastatic crosstalk between melanoma and lymphatic endothelial cells [36].

Notch signaling plays an important role in the progression and metastasis of various human cancers. In this study, we demonstrated for the first time endogeneous interactions between Notch2/3 and the ligand DLL4 as well as a strong positive correlation between Notch2/3 expression and the expression levels of the canonical Notch downstream targets HES1 and HEY1. Addition of exogeneous DLL4 resulted in enhanced tumor cell numbers via Notch2 and/or Notch3-mediated canonical pathway activation. In conclusion, our results support the view that the Notch2/3-DLL4 axis play an oncogenic role in BCa.

### Limitations

Many questions about the molecular and cellular effects of Notch signaling in BCa remain to be answered.

1. Our study was realized using 2D-cultured BCa cell lines. These in vitro models do not fully reflect the biological diversity of bladder tumors. Future research should focus on the role of the Notch2/3-DLL4 axis in BCa organoids consisting of different cell types, which better recapitulate disease heterogeneity in solid tumors. Studies in BCa organoids would enable more reliable analyses of cellular Notch crosstalk via cis- and/or trans-activation between tumor cells, fibroblasts and endothelial cells.
2. Gene expression data of BCa cell lines showed moderate to high correlation with primary BCa tissue expression data, but results should be handled carafully. Expression data extracted from TCGA database are based on bulk RNA sequencing which does not take any cell type-specific expression patterns into account.
3. We were able to demonstrate co-localization of Notch2/3 and DLL4 by PLA. However, it remains unclear wether these findings are merely based on the spatial proximity of the targets or wether it is actually a proof of direct protein-protein-interactions.
4. DLL4 can activate both canonical and non-canonical Notch2/3 signaling, but it is still unclear which pathway plays the essential role in BCa. It is noteworthy that studying the function of Notch signaling requires combinatorial deletion/overexpression of Notch pathway members such as Notch receptors and ligands.

## Supplementary material

S1 Methods Detailed protocols for qRT-PCR, immunofluorescence, in situ PLA and DLL4 stimulation. (DOCX)

S1 Table Quantification data and significances of all findings presented in the main manuscript. (XLSX)

S1 Fig Bioinformatics data analysis. Results validation by analyzing gene expression data from open-source databases. (A and B) Heatmap of target gene expression levels in different cell lines from CCLE datasets (A) versus data from the present study (B). Gene expression data of Notch family members were downloaded from cancer cell line encyclopedia (CCLE, https://portals.broadinstitute.org/ccle, cited 2021 Feb 28); colors encode the average value of mRNA expression levels. (C and D) Correlation of gene expression between BCa cell lines and primary BCa tumors. Target gene expression data for bladder cancer samples (TCGA-BLCA) were downloaded from The Cancer Genome Atlas (TCGA, https://www.cancer.gov/tcga; cited 2021 Feb 27). CCLE data sets and TCGA database were analyzed for correlation. Statistical software R (Version 4.0.2. https://www.r-project.org/) with R studio (Version 1.2.5042) was utilized. (C) Graphic representation of Pearson correlation coefficients demonstrate moderate to high correlation of the examined cell lines with primary tissue; (B) hierarchical clustering based on correlation.
(TIF)

S2 Fig Subcellular localization of DLL4 and Notch2/3 in low-grade BCa cell lines. Representative images of double immunofluorescence images of simultaneously stained Notch2/3 and DLL4 in low-grade BCa cells (LSM800; 63x oil immersion objective; pinhole 47 µm; 1AU; 0.9 µm opical section). While Notch2/3 and DLL4 were diffusely distributed throughout the cytoplasm of 647-V cells, Notch proteins in RT-112 showed a cytoplasmic (DLL4, Notch3), perinuclear (DLL4, Notch2) or even nuclear localization (Notch3). In RT-4, DLL4 showed a major cytoplasmic localization; Notch2 was abundant in the cytoplasm and the perinuclear region; Notch3 was detected in the cytoplasm or at the cell membrane. Scale bar: 10 µm.
(TIF)

S3 Fig Subcellular localization of DLL4 and Notch2/3 in high-grade BCa cell lines. Representative images of double immunofluorescence images of simultaneously stained Notch2/3 and DLL4 in high-grade BCa cells (LSM800; 63x oil immersion objective; pinhole 47 µm; 1AU; 0.9 µm opical section). While Notch2/3 and DLL4 were diffusely distributed throughout KU-19-19 cells, Notch proteins showed a predominantly perinuclear (DLL4, Notch2) or even nuclear localization (Notch3) in T-24 and CAL-29. Scale bar: 10 µm.
(TIF)

S4 Fig Correlation of Notch2/3-DLL4 interaction complexes with cell line grading. (A) No correlation of total number of Notch2/3-DLL4 with tumor grade; (B) significant correlation of (peri-) nuclear Notch2/3-DLL4 with grade; * p ≤ 0.05; Pearson correlation analysis; n=3.
(TIF)

S5 Fig Subcellular localization of PLA signals and PLA negative controls. (A) Subcellular localization of Notch2/3-DLL4 PLA signals; exemplarily shown for RT-4 and CAL-29 cells. Increased optical sectioning sharpness (63 x/1.4 NA oil immersion objective; pinhole: 36 µm; 0.68 AU; 0.7 µm optical section) verified a predominately membraneous and cytoplasmic localization of Notch2/3-DLL4 in RT-4, while Notch2/3 was predominately found in the perinuclear region (arrows) in CAL-29 cells. Scale bar: 10 µm. (B) PLA negative controls. Images of technical negative control staining, exemplarily shown for RT-4, T-24 and KU-19-19 cells. PLA controls were performed by incubation with only one primary antibody, followed by treatment with PLA probe mixture and PLA detection reagents.
(TIF)

S6 Fig Correlation of downstream targets HES1 and HEY1 with Notch2/3-DLL4 receptor complexes. (A) Correlation of total number of Notch2/3-DLL4 with HES1/HEY1 protein expression; (B) negative correlation of (peri-) nuclear Notch3-DLL4 with HES1/HEY1 protein expression; * p ≤ 0.05; Pearson correlation analysis; n=3.
(TIF)

S7 Fig Migration of BCa cells in response to recombinant human DLL4 (rhDLL4). Cells were cultured for 72 h in 96-well plates until confluency was reached. The Incucyte^®^ 96-Well Woundmaker Tool (Sartorius, Epsom, UK) was used for wounding procedure to create precise and reproducible wounds in all monolayers. After wounding, medium was aspirated from each well and wells were gently washed two times with culture medium to prevent dislodged cells from settling and reattaching. After washing, 100 µl medium containing rhDLL4 (250, 500, 1000 ng/ml) was added. Plates were placed into the Incucyte^®^ Live-Cell Analysis System (Sartorius, Epsom, UK). Repeated scanning every 2 h for 68 h was scheduled for live-cell imaging. Quantification of relative wound density (%) was automatically performed using the Incucyte^®^ Software: Scratch Wound Cell Migration and Invasion Analysis Metrics (Sartorius, Epsom, UK). Relative wound density is a measure (%) of the density of the wound region relative to the density of the cell region. Migration assays indicated induction of increased wound density during the first 24 h in T-24, KU-19–19, and during 68 h in RT-4; although it was not significant due to high standard deviations. Mean + SD.
(TIF)
