# Supplementary material for: Notch2/3-DLL4 interaction in urothelial cancer cell lines supports a tumorigenic role of Notch signaling pathways in bladder carcinoma
Source: PLoS One. 2025 Feb 14;20(2):e0317709. doi: 10.1371/journal.pone.0317709 (PMC11828355; doi:10.1371/journal.pone.0317709)
Supplement: S1 Methods — (DOCX) [file pone.0317709.s001.docx]

**Supporting information: Methods S1**

**RNA extraction and qRT-PCR**

**RNA extraction**

- Kit: RNeasy® Plus Micro Kit (Cat.-No.: 74034, Qiagen, Hilden, Germany)
- Cells (5 x 10^5^) were directly lyzed in the cell culture dish by addition of 350 µl buffer RLT Plus
- Lysates were transferred to a gDNA Eliminator column placed in a 2 ml collection tube
- Centrifugation for 30 s at ≥8000 x g (flow-through was saved)
- Same volume of 70% ethanol was added to the flow-through and mixed by pipetting
- Samples were transferred to an RNeasy MinElute spin column placed in a 2 ml collection tube; centrifugation for 15 s at ≥8000 x g (flow-through was discarded)
- Buffer RW1 (700 µl) was added to the column; centrifugation for 15 s at ≥8000 x g (flow-through was discarded)
- Buffer RPE (500 µl) was added to the column; centrifugation for 15 s at ≥8000 x g (flow-through was discarded)
- 80% ethanol (500 µl) was added to the column; centrifugation for 2 min at ≥8000 x g (flow-through was discarded)
- RNeasy MinElute spin column was placed in a new 2 ml collection tube and centrifuged at full speed to dry the membrane
- RNeasy MinElute spin column was placed in a new 1.5 ml collection tube; RNase-free water was added directly to the center of the spin column; centrifugation for 1 min at full speed to elute RNA

**cDNA synthesis**

- Kit: Maxima First Strand cDNA Synthesis Kit for RT-qPCR (Cat.-No.: K1641, Thermo Fisher Scientific, Dreieich, Germany)
- The following components were added in the indicated order into a sterile, RNase-free tube on ice:
  - 5 x Reaction Mix 4 µl
  - Maxima Enzyme Mix 2 µl
  - Template RNA 1 µg
  - Water, nuclease-free to 20 µl
- Incubation for 10 min at 25 °C followed by 15 min at 50 °C
- Termination of the reaction by heating at 85 °C for 5 min

**qRT-PCR**

- Kit: Maxima SYBR Green/ROX qPCR Master Mix (2X) (Cat.-No.: K0221, Thermo Fisher Scientific, Dreieich, Germany)
- Preparation of a reaction master mix by adding the following components (except template DNA) for each 25 μl reaction to a tube at room temperature:
  - Maxima SYBR Green/ROX qPCR Master Mix (2X) 12.5 µl
  - Forward Primer 0.3 µM
  - Reverse Primer 0.3 µM
  - Template DNA 50 ng
  - Water, nuclease-free to 20 µl
- Mixing and centrifugation
- Three-step thermal cycling program:

| Step | Temperature, °C | Time | Number of cycles |
| --- | --- | --- | --- |
| UDG pre-treatment | 50 | 2 min | 1 |
| Initial denaturation | 95 | 10 min | 1 |
| Denaturation | 95 | 15 s | 40 |
| Annealing | Primer-specific | 30 s |  |
| Extension | 72 | 30s |  |

- Data acquisition was performed during the extension step

**Immunofluorescence**

- Cells (50.000 cells per well) were cultured on Collagen A-coated coverslips in a 24-well plate for 24 h
- Cells were washed (1 x 5 min) with PBS (with Ca, Mg) at room temperature
- Cells were fixed with ice-cold methanol for 15 minutes at 4°C
- Samples were washed (3 x 5 min) with PBS at room temperature
- Samples were washed (1 x 5 min) with TBS at room temperature
- Samples were permeabilized (5 min) with 0.3% DMSO + 0.1% Triton X-100 in TBS
- Incubation of samples for 15 min (room temperature) with blocking solution: 3% milk powder + 1% BSA in TBS
- Incubation of samples with primary antibodies (diluted in blocking solution) overnight at 4°C
- Samples were washed (1 x 5 min) with TBS at room temperature
- Incubation of samples with secondary antibodies (1:500, diluted in TBS) for 1 h at room temperature
- Samples were washed (1 x 5 min) with TBS at room temperature
- Incubation of samples with Alexa Fluor 488®/555®-coupled secondary antibodies (Thermo Fisher Scientific, Dreieich, Germany) (1:500, diluted in TBS) for 1 h at room temperature
- Samples were washed (1 x 5 min) with TBS at room temperature
- Nuclear staining with TO-PRO-3 iodide (Thermo Fisher Scientific, Dreieich, Germany) (1:1000, diluted in TBS) for 20 min at room temperature
- Samples were washed (1 x 5 min) with TBS at room temperature
- Mounting of samples with Confocal-Matrix® (Micro-Tech Lab, Graz, Austria)
- Storage of slides protected from light at 4°C

**In situ PLA**

**Preparation of wash buffers**

- PLA In situ wash buffer A: 0.01 M Tris, 0.15 M NaCl and 0.05% Tween 20 in high purity water; filtration through a 0.22 µm filter and storage at 4°C
- PLA In situ wash buffer A: 0.2 M Tris and 0.1 M NaCl in high purity water; filtration through a 0.22 µm filter and storage at 4°C

**Antibody labeling**

- Cells (50.000 cells per well) were cultured on Collagen A-coated coverslips in a 24-well plate for 24 h
- Cells were washed (1 x 5 min) with PBS (with Ca, Mg)
- Cells were fixed with 4% formaldehyde for 30 minutes at 4°C
- Samples were washed (3 x 5 min, shaking) with PBS
- Samples were washed (1 x 5 min, shaking) with TBS
- Samples were permeabilized (5 min) with 0.3% DMSO + 0.1% Triton X-100 in TBS
- Incubation of samples with blocking solution: 3% milk powder + 1% BSA in DMSO/Triton X-100 solution
- Incubation of samples with primary antibodies (diluted in blocking solution) overnight at 4°C

**Detection**

- Samples were washed (1 x 5 min) with TBS
- PLA probes: Dilution of Duolink® In situ PLA probes (PLUS/MINUS; Sigma-Aldrich, Munich, Germany) depending on primary antibody species (1:5 in blocking solution); pre-incubation for 20 min at room temperature
- Samples were washed (1 x 10 min) with TBS
- Incubation of samples with PLA probe mixture for 1 h at 37°C on a orbital shaker
- Samples were washed (2 x 5 min) with PLA wash buffer A at room temperature on a orbital shaker
- Ligation: Dilution of Ligation Mix (Duolink® In situ Detection Kit; Sigma-Aldrich, Munich, Germany) 1:5 in nuclease-free water (mix); addition of ligase enzyme with 1:40 dilution to ligation mixture (mix); incubation of samples with ligation mixture for 30 min at 37°C on a orbital shaker
- Samples were washed (2 x 5 min) with PLA wash buffer A at room temperature on a orbital shaker
- The following steps were performed protected from light
- Amplification: Dilution of Amplification Mix (Duolink® In situ Detection Kit; Sigma-Aldrich, Munich, Germany) 1:5 in nuclease-free water (mix); addition of polymerase enzyme with 1:80 dilution to amplification mixture (mix); incubation of samples with amplification mixture for 100 min at 37°C on a orbital shaker
- Samples were washed (2 x 10 min) with PLA wash buffer B at room temperature on a orbital shaker
- Cell membrane labeling (for ROI selection in Image J): Dilution of wheat germ agglutinin (WGA, Alexa Fluor® 488-labeled; Thermo Fisher Scientific, Dreieich, Germany) 1:200 in PLA wah buffer B (mix); incubation of samples with WGA for 20 min at room temperature on a orbital shaker
- Samples were washed (2 x 5 min) with PLA wash buffer B at room temperature on a orbital shaker
- Samples were washed (1 x 5 min) with 0.01 x PLA wash buffer B at room temperature on a orbital shaker
- Coverslips were dried for 15 min in the dark
- Mounting of samples with Duolink® In situ Mounting medium with DAPI (Sigma-Aldrich, Munich, Germany)
- Storage of slides protected from light at -20°C

**DLL4 treatment response**

**Cell viability assay**

- Kit: CellTiter-Glo® Luminescent Cell Viability Assay (Cat.-No.: G7570, Promega, Mannheim, Germany)
- Cells were cultured in 96 well plates (white, clear bottom) and treated with recombinant human DLL4 (diluted in culture medium) for 72 h; DLL4 was replaced every 24 h
- For viability assay, DLL4 was replaced by 50 µl culture medium
- Preparation of control wells containing medium without cells to obtain a value for background luminescence
- Culture plates were equilibrated at room temperature for approximately 30 min
- CellTiter-Glo® Reagent (50 µl) was added cell culture medium present in each well
- Samples were mixed 2 min on an orbital shaker to induce cell lysis
- Incubation at room temperature for 10 min to stabilize the luminescent signal
- Measurement of luminescence using a SpectraMax M5 microplate reader

**Cell proliferation assay**

- Kit: CellTiter 96® Non-Radioactive Cell Proliferation Assay (Cat.-No.: G4000, Promega, Mannheim, Germany)
- Cells were cultured in 96 well plates (black, clear bottom) and treated with recombinant human DLL4 (diluted in culture medium) for 72 h; DLL4 was replaced every 24 h
- For proliferation assay, DLL4 was replaced by 100 µl culture medium
- Preparation of control wells containing medium without cells to obtain a value for background luminescence
- Addition of 15µl Dye Solution to each well
- Incubation of plates at 37°C for 4 h in a humidified CO2 incubator
- Addition of 100µl Solubilization/Stop Solution to each well
- Measurement of absorbance at 570nm using a SpectraMax M5 microplate reader
