# Supplementary figures and images for: Notch2/3-DLL4 interaction in urothelial cancer cell lines supports a tumorigenic role of Notch signaling pathways in bladder carcinoma
Source: PLoS One. 2025 Feb 14;20(2):e0317709. doi: 10.1371/journal.pone.0317709 (PMC11828355; doi:10.1371/journal.pone.0317709)

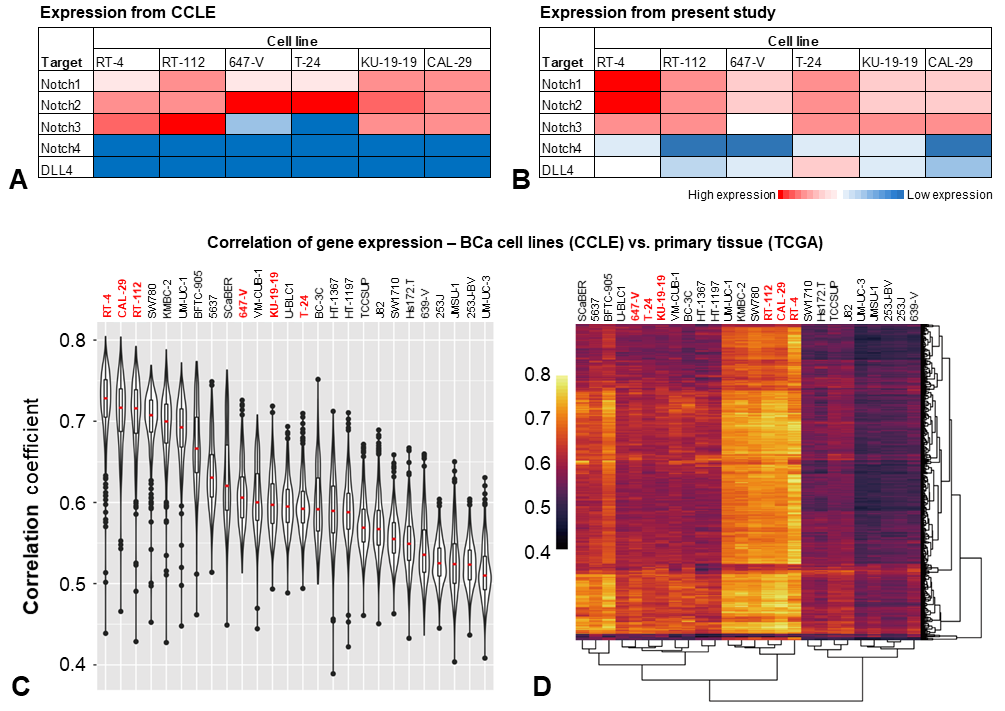

Supplement: S1 Fig — (A and B) Heatmap of target gene expression levels in different cell lines from CCLE datasets (A) versus data from the present study (B). Gene expression data of Notch family members were downloaded from cancer cell line encyclopedia (CCLE, https://portals.broadinstitute.org/ccle, cited 2021 Feb 28); colors encode the average value of mRNA expression levels. (C and D) Correlation of gene expression between BCa cell lines and primary BCa tumors. Target gene expression data for bladder cancer samples (TCGA-BLCA) were downloaded from The Cancer Genome Atlas (TCGA, https://www.cancer.gov/tcga; cited 2021 Feb 27). CCLE data sets and TCGA database were analyzed for correlation. Statistical software R (Version 4.0.2. https://www.r-project.org/) with R studio (Version 1.2.5042) was utilized. (C) Graphic representation of Pearson correlation coefficients demonstrate moderate to high correlation of the examined cell lines with primary tissue; (B) hierarchical clustering based on correlation. (TIF) [file pone.0317709.s003.tif]

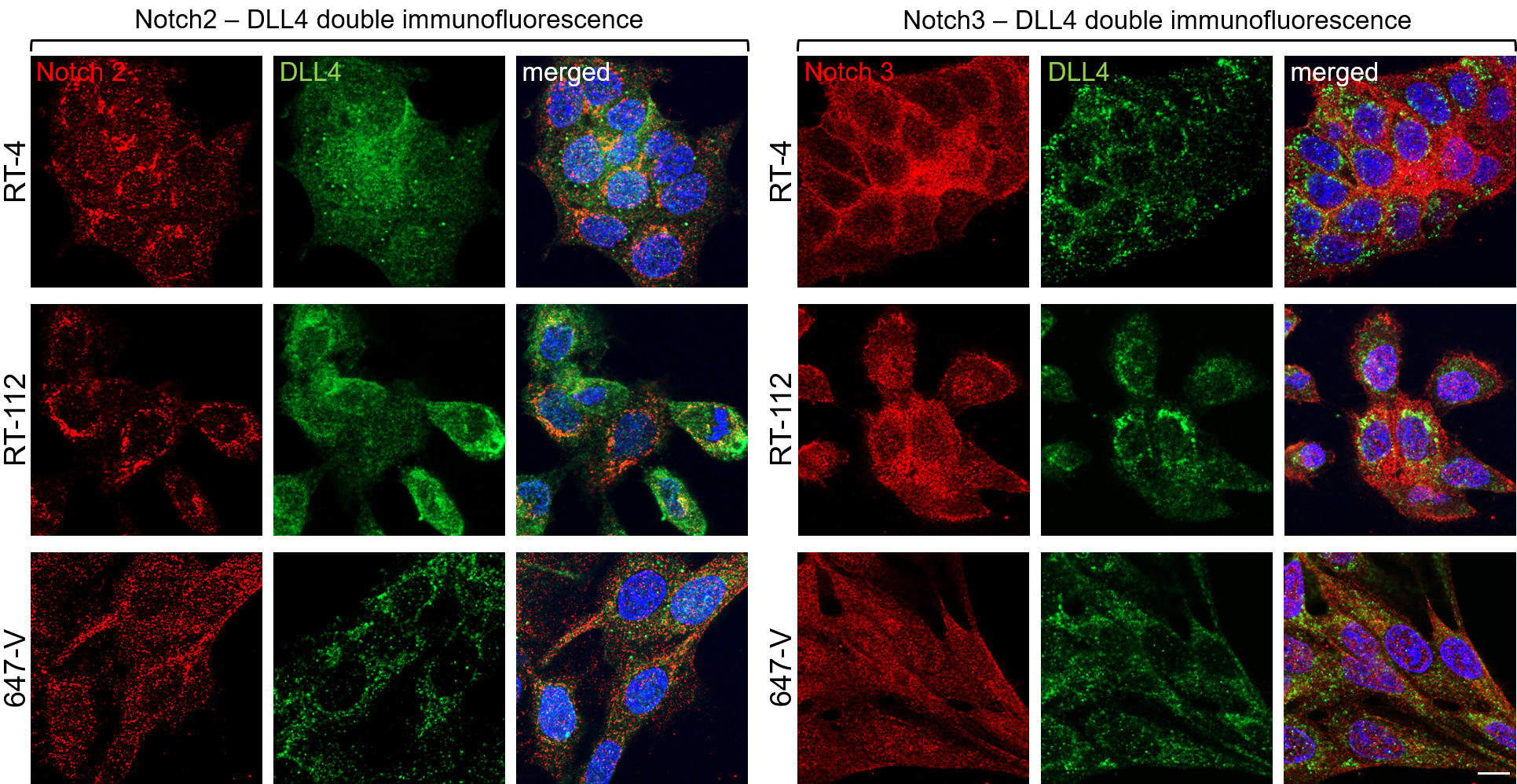

Supplement: S2 Fig — Representative images of double immunofluorescence images of simultaneously stained Notch2/3 and DLL4 in low-grade BCa cells (LSM800; 63x oil immersion objective; pinhole 47 µm; 1AU; 0.9 µm opical section). While Notch2/3 and DLL4 were diffusely distributed throughout the cytoplasm of 647-V cells, Notch proteins in RT-112 showed a cytoplasmic (DLL4, Notch3), perinuclear (DLL4, Notch2) or even nuclear localization (Notch3). In RT-4, DLL4 showed a major cytoplasmic localization; Notch2 was abundant in the cytoplasm and the perinuclear region; Notch3 was detected in the cytoplasm or at the cell membrane. Scale bar: 10 µm. (TIF) [file pone.0317709.s004.tif]

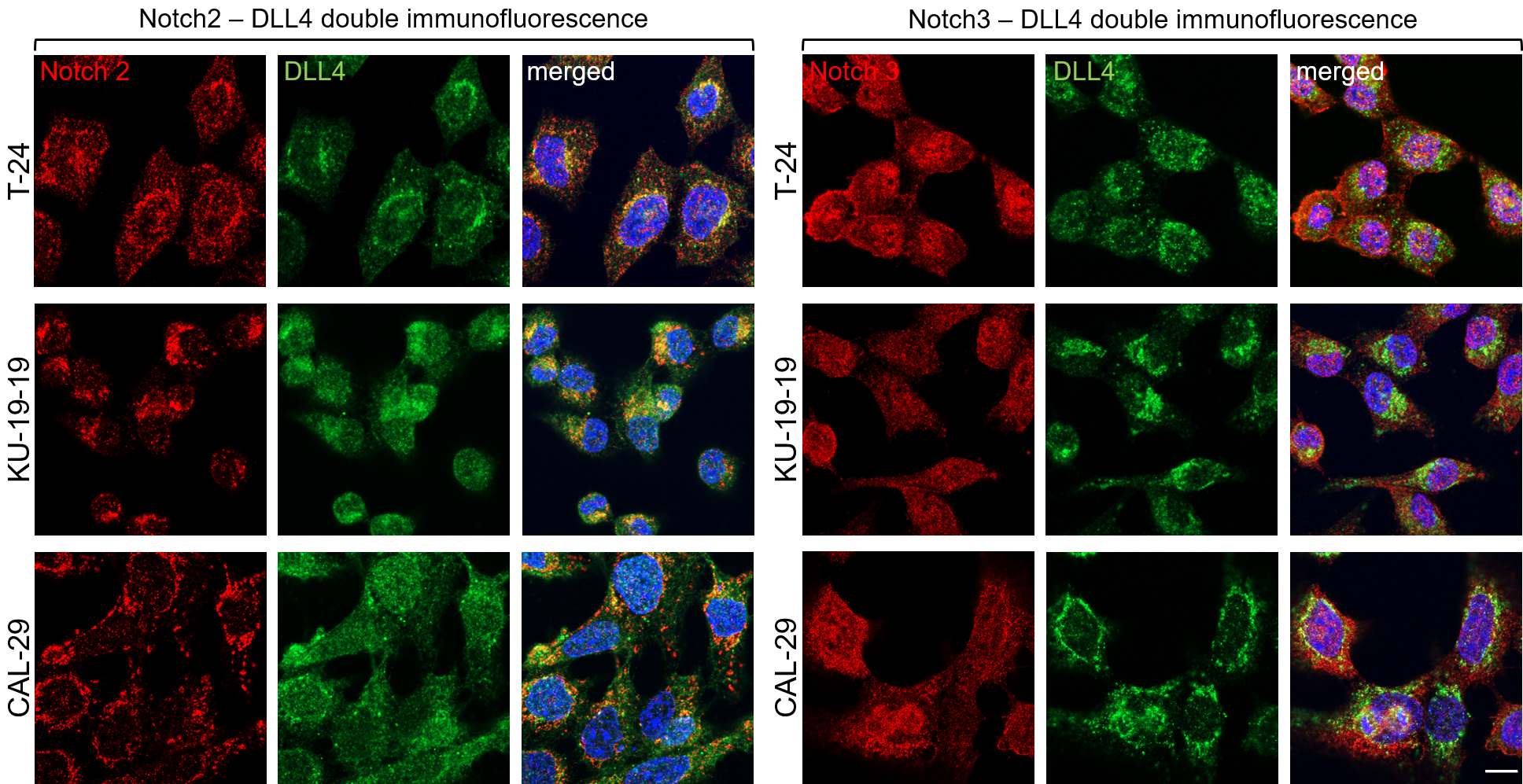

Supplement: S3 Fig — Representative images of double immunofluorescence images of simultaneously stained Notch2/3 and DLL4 in high-grade BCa cells (LSM800; 63x oil immersion objective; pinhole 47 µm; 1AU; 0.9 µm opical section). While Notch2/3 and DLL4 were diffusely distributed throughout KU-19-19 cells, Notch proteins showed a predominantly perinuclear (DLL4, Notch2) or even nuclear localization (Notch3) in T-24 and CAL-29. Scale bar: 10 µm. (TIF) [file pone.0317709.s005.tif]

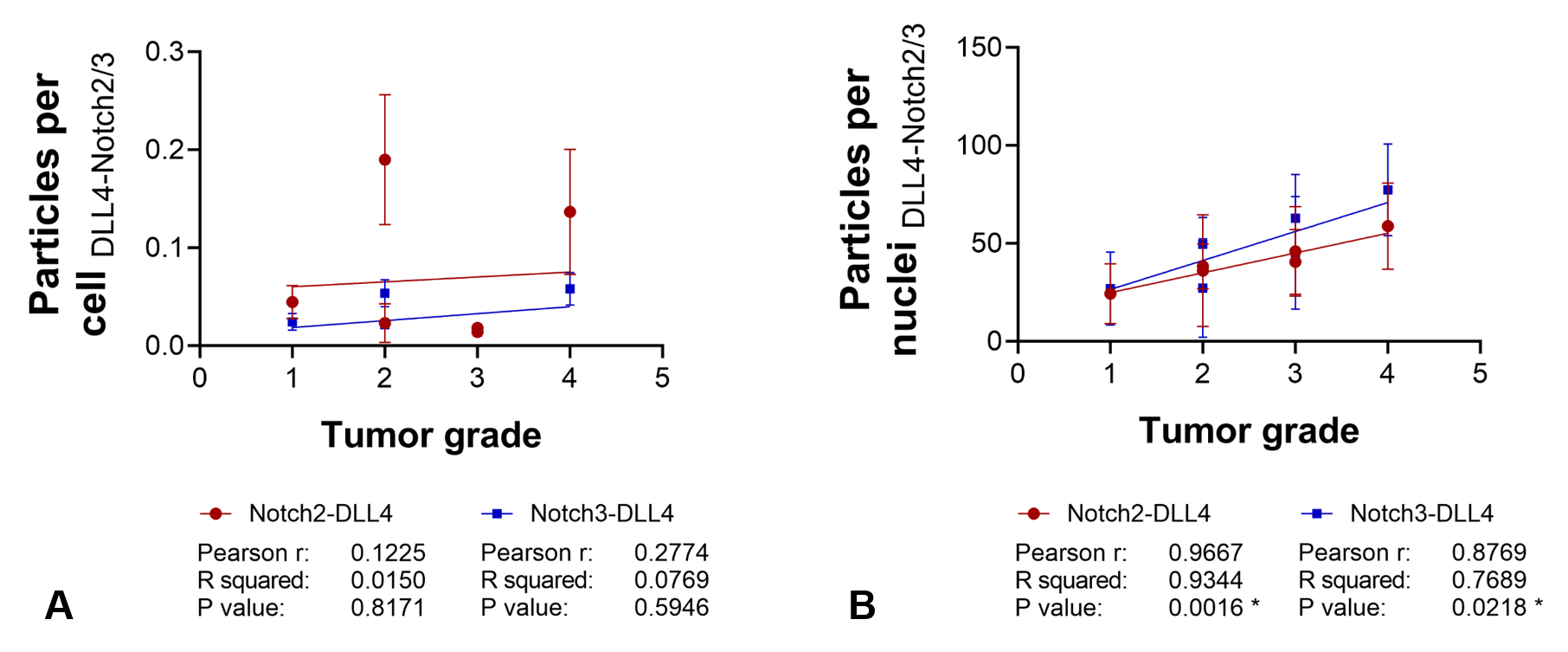

Supplement: S4 Fig — (A) No correlation of total number of Notch2/3-DLL4 with tumor grade; (B) significant correlation of (peri-) nuclear Notch2/3-DLL4 with grade; * p ≤ 0.05; Pearson correlation analysis; n=3. (TIF) [file pone.0317709.s006.tif]

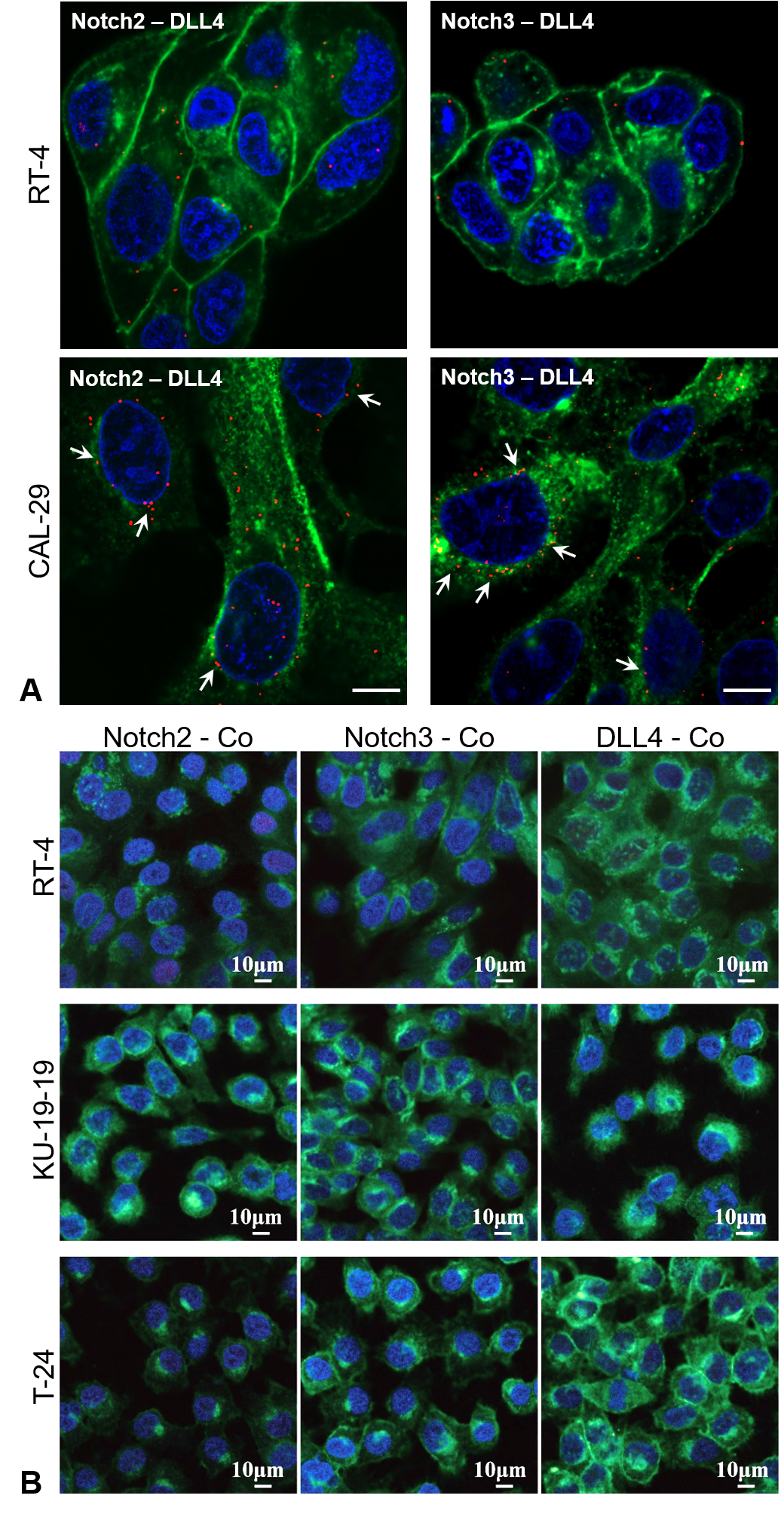

Supplement: S5 Fig — (A) Subcellular localization of Notch2/3-DLL4 PLA signals; exemplarily shown for RT-4 and CAL-29 cells. Increased optical sectioning sharpness (63 x/1.4 NA oil immersion objective; pinhole: 36 µm; 0.68 AU; 0.7 µm optical section) verified a predominately membraneous and cytoplasmic localization of Notch2/3-DLL4 in RT-4, while Notch2/3 was predominately found in the perinuclear region (arrows) in CAL-29 cells. Scale bar: 10 µm. (B) PLA negative controls. Images of technical negative control staining, exemplarily shown for RT-4, T-24 and KU-19-19 cells. PLA controls were performed by incubation with only one primary antibody, followed by treatment with PLA probe mixture and PLA detection reagents. (TIF) [file pone.0317709.s007.tif]

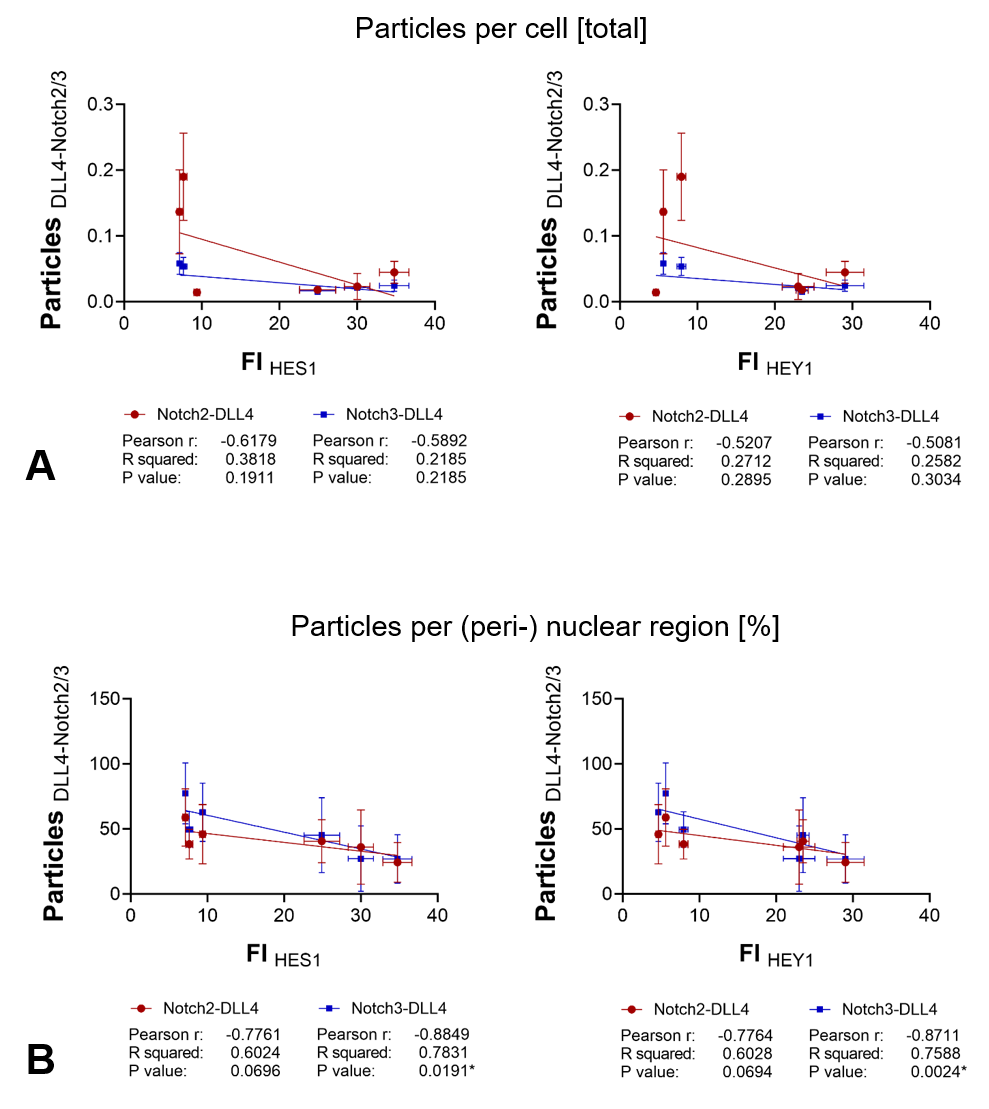

Supplement: S6 Fig — (A) Correlation of total number of Notch2/3-DLL4 with HES1/HEY1 protein expression; (B) negative correlation of (peri-) nuclear Notch3-DLL4 with HES1/HEY1 protein expression; * p ≤ 0.05; Pearson correlation analysis; n=3. (TIF) [file pone.0317709.s008.tif]

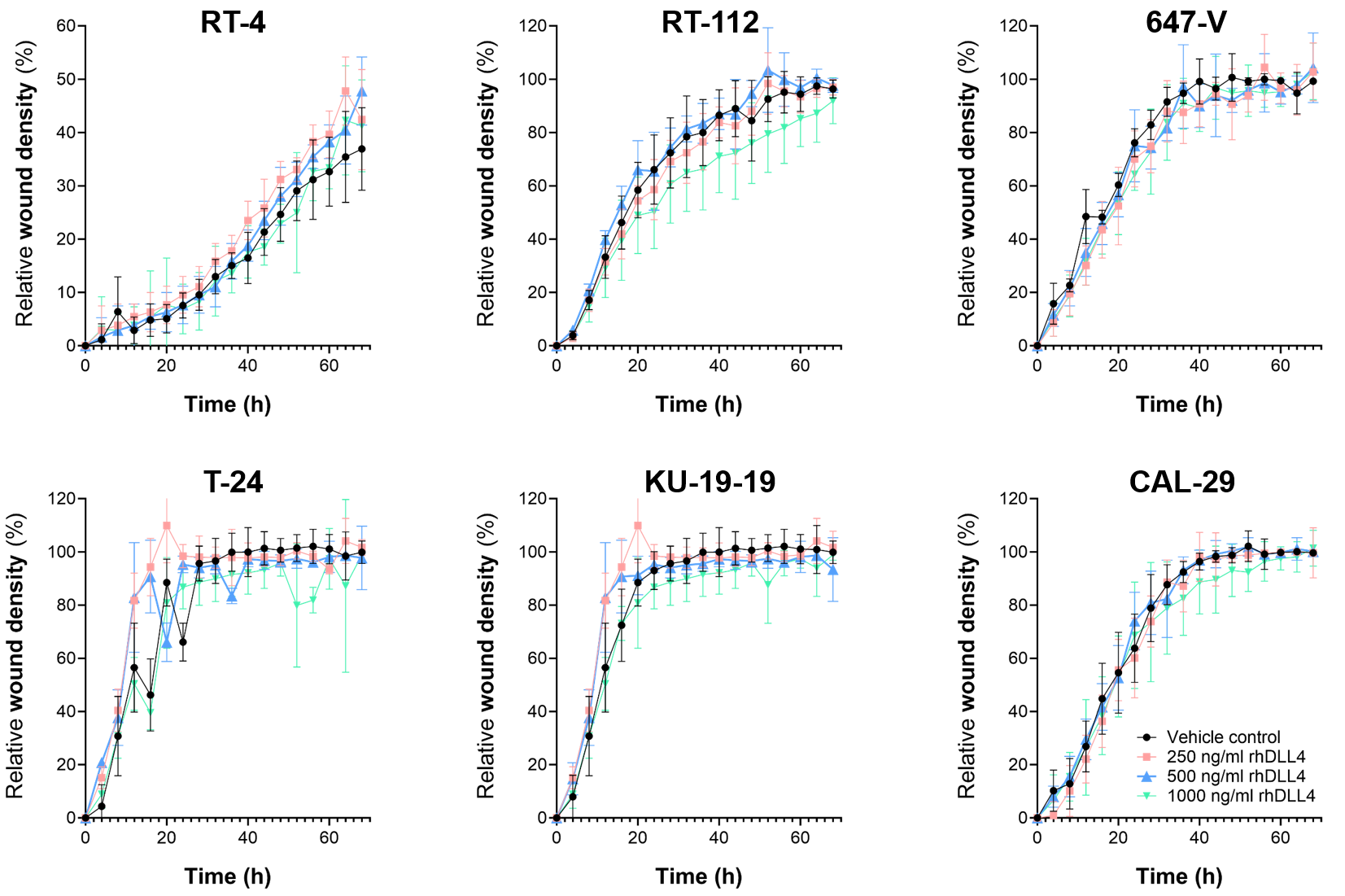

Supplement: S7 Fig — Cells were cultured for 72 h in 96-well plates until confluency was reached. The Incucyte® 96-Well Woundmaker Tool (Sartorius, Epsom, UK) was used for wounding procedure to create precise and reproducible wounds in all monolayers. After wounding, medium was aspirated from each well and wells were gently washed two times with culture medium to prevent dislodged cells from settling and reattaching. After washing, 100 µl medium containing rhDLL4 (250, 500, 1000 ng/ml) was added. Plates were placed into the Incucyte® Live-Cell Analysis System (Sartorius, Epsom, UK). Repeated scanning every 2 h for 68 h was scheduled for live-cell imaging. Quantification of relative wound density (%) was automatically performed using the Incucyte® Software: Scratch Wound Cell Migration and Invasion Analysis Metrics (Sartorius, Epsom, UK). Relative wound density is a measure (%) of the density of the wound region relative to the density of the cell region. Migration assays indicated induction of increased wound density during the first 24 h in T-24, KU-19–19, and during 68 h in RT-4; although it was not significant due to high standard deviations. Mean + SD. (TIF) [file pone.0317709.s009.tif]
